# Overview of Risk Factors and Diagnosis of Invasive Candidiasis

**DOI:** 10.3390/jof12060383

**Published:** 2026-05-25

**Authors:** Valentina Daniela Sisu, Anda Băicuș

**Affiliations:** Faculty of Medicine, University of Medicine and Pharmacy, Carol Davila, 020021 Bucharest, Romania

**Keywords:** risk factors, candidemia, intra-abdominal candidiasis, diagnostic algorithm, prediction models

## Abstract

Invasive candidiasis is a significant concern in healthcare environments, and awareness of these infections has increased in recent years. A growing number of risk factors, the ability of some *Candida* species to progress from colonization to tissue invasion, and their capacity to adhere to and survive on abiotic surfaces have all contributed to the spread of invasive candidiasis. The primary goal in cases of invasive candidiasis is to diagnose it as promptly as possible, as any delay can delay antifungal treatment. This review concentrates on clinical syndromes reunited under the definition of invasive candidiasis and the current diagnostic methods. Risk factor assessment is another major topic of this narrative review and recent updates are included. Research stage biomarkers are also explored and partial results are mentioned as there are continuous efforts to search for new tools for a more accurate prediction or an earlier identification of IC.

## 1. Introduction

Once considered rare, fungal infections are now an emerging global threat to public health because of their ability to cause serious systemic infections in immunocompromised patients. Most cases are caused by *Candida* species, followed by *Aspergillus* species, *Cryptococcus* species, and *Pneumocystis* species.

Candidiasis is a broad term that covers both superficial and invasive infections. Superficial candidiasis refers to skin infections that primarily occur in moist, warm areas of the skin, usually with mild symptoms, and also includes mucosal infections.

Invasive candidiasis involves deep tissue infections and bloodstream infections, with the latter also known as candidemia. These can occur independently or often as part of a related sequence of events. The development of candidemia creates a vicious cycle that eventually leads to secondary deep-seated infections. The incidence of candidemia has increased in recent years, both in Intensive Care Unit (ICU) and non-ICU patients, due to population aging, exposure to a greater number of invasive procedures, novel immunosuppressive therapies, the use of broad-spectrum antibiotics and, of course, because of the faster spread of *Candida* spp. and increased opportunity to colonize and invade tissues [1].

Early diagnosis and management of invasive candidiasis is challenging but crucial for improving prognosis.

This narrative review focuses on the following: (1) the risk factors for occurrence of invasive candidiasis (IC), (2) microbiological and serological diagnosis of IC, (3) IC clinical predictive models and (4) candidate biomarkers and their current state of research. The aim of this review is to provide a clinician-oriented overview of the main categories of invasive candidiasis with the current definitions and the commercially available diagnostic tools. It also provides recent updates to the basic knowledge on risk factors, and their relative weight in the occurrence of IC. The review also summarizes the current prediction models, highlighting characteristics that are relevant for clinicians. The role of these models in identifying risk categories and assisting in treatment is also emphasized. The review contains a final section dedicated to research stage biomarkers, providing information about the detection techniques and partial results in terms of sensitivity and specificity.

## 2. Epidemiology

Over recent decades, the prevalence of invasive fungal infections has increased in healthcare settings. *Candida* species are the fourth most common pathogens in bloodstream infections in the U.S., especially among hospitalized patients in intensive care units.

Active surveillance for culture-confirmed candidemia conducted by the CDC through the Emerging Infections Program (EPI) identified an incidence rate of 8.7 per 100,000 people from 2012 to 2016. Similarly, in Europe, candidemia remains a significant concern, ranking between 6th and 10th among causes of bloodstream infections [2]. A comprehensive meta-analysis of population-based studies across European countries over two decades found an incidence of 3.88 cases per 100,000 people per year, with most cases reported in ICUs (5.5 per 1000) [3]. A more recent comprehensive meta-analysis conducted by Denning et al. estimates an annual incidence of 626,081 cases of candidemia after collecting data from papers published between 2010 and 2023 from 98 countries [4].

The EUCANDICU project involved 23 ICUs across nine European countries and found an incidence of 7.07 ICU-acquired invasive candidiasis episodes per 1000 ICU admissions. This is the largest multicenter, multinational retrospective study to evaluate invasive candidiasis in ICU settings. It also reported a crude 30-day mortality rate of approximately 42%. The most common isolate was *Candida albicans* at 57%, followed by *Candida glabrata* at 21%, and *Candida parapsilosis* at 13% [5].

In a multicenter retrospective study, Meyer et al. reported a 1-year cumulative incidence of invasive candidiasis of 2.8% in solid organ transplant recipients. The median time between transplantation and invasive candidiasis occurrence was 136 days and candidemia was less frequently encountered (23.3%) when compared to all intra-abodominal candidiasis cases (gastrointestinal tract—41.2%, liver/biliary tract—9.8%). The IC diagnosed in the first year after transplant had an impact on mortality in all solid organ transplant recipients. A higher 12-week all-cause mortality rate was observed in candidemia (51.1%) compared with intra-abdominal (14%) and other IC sites (17.3%) [6]. In a meta-analysis, Popova et al. reported an incidence of 5.22% of invasive fungal diseases in oncohematological patients that receive chemotherapy and a 9.96% incidence in patients undergoing allogeneic hematopoietic stem cell transplantation. Pathogen distribution was dominated by *Candida* spp. and *Aspergillus* spp. in both oncohematological patients (*Candida* spp.—39.55%, *Aspergillus* spp.—35.98%) and allo-HSCT patients (*Candida* spp.—17.28%, *Aspergillus* spp.—66.11%) [7].

The global crude mortality rate in *Candida* bloodstream infections is high, reaching 35% in treated cases, and almost 90% in untreated cases. The percentage of deaths attributable to this is 60% [4]. Mortality percentages differ within invasive candidiasis categories, with the mortality rate for transient candidemia (e.g., secondary to catheter use) being significantly lower than that for candidemia caused by a deep focus (e.g., intra-abdominal infections) [8].

## 3. Risk Factors

Increased colonization, along with weakened local or systemic defenses, creates perfect conditions for infection. Factors like microbiome imbalance due to immunosuppression, long-term antibiotic use, or mucosal barrier damage from gastrointestinal surgery, chemotherapy, or inflammation are common contributors [9].

However, the source of invasive candidiasis is not solely endogenous. It can also originate from external sources, such as contaminated solutions, materials, endotracheal tubes, catheters, central lines, shunts, stents, and other intravascular devices. Another reservoir for some *Candida* spp. is the skin microbiota of healthcare workers, which can transmit the fungi to patients. After inoculation, these fungi can survive for up to 45 min on skin and nearly 4 months on hospital surfaces. *Candida* spp. resistance in the hospital setting is heightened by their ability to form biofilms on inert surfaces.

### 3.1. Major Risk Factors

**Figure 1:**
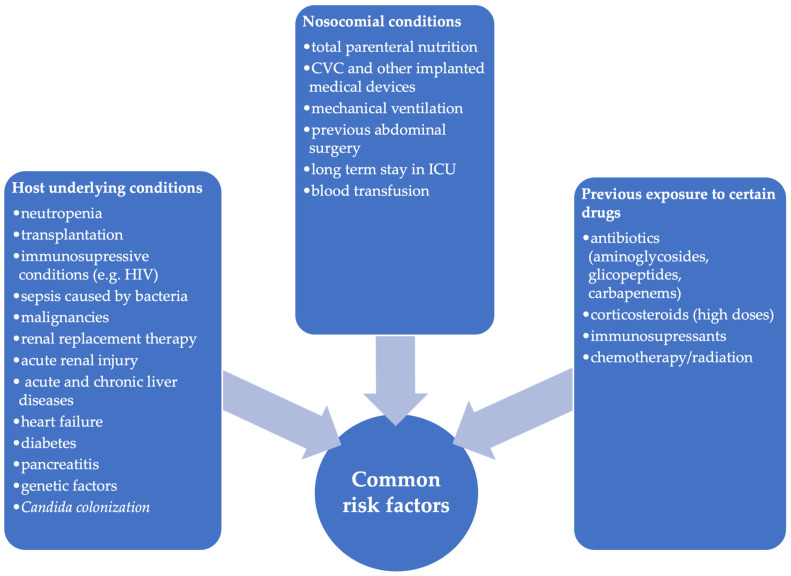
Risk factors for invasive candidiasis and candidemia in adult patients (references: [10,11,12,13,14]).

In multicenter matched case–control studies the common risk factors in the ICU were total parenteral nutrition, prolonged hospital stays, previous abdominal surgery, acute kidney injury, hemodialysis, heart disease, previous septic shock and exposure to aminoglycosides (Figure 1).

For non-ICU patients, central venous catheters, total parenteral nutrition, and exposure to glycopeptides and nitroimidazoles were the significant risk factors for candidemia. The specificity of risk factors for these separate groups is determined by specific patterns of antibiotic exposure, clinical features and medical equipment [10,11,12].

Parenteral nutrition is recognized as a major independent risk factor for candidemia, involving several potential mechanisms. Key factors include atrophy of the intestinal mucosa, which can lead to the translocation of microorganisms or endotoxins into the bloodstream, hyperglycemia, and the use of parenteral access devices. Moreover, improper preparation, storage, or handling of nutrition solutions can result in contamination with *Candida* spp., increasing the risk of infection. Certain components may also promote microbial growth; for example, fat emulsions serve as excellent media for bacterial and fungal species. Also, lipid-containing solutions can support biofilm formation and the germination of *Candida* spp. The most common infections associated with parenteral nutrition are catheter-related bloodstream infections, often caused by migration of the skin’s native microflora or contamination of the infusate or catheter hub [13].

Poissy et al. found that central venous catheters were an independent risk factor for candidemia in non-ICU patients rather than ICU patients, probably because they are very frequently used (>90% of patients) in the ICU, and thus they become non-discriminant for the determination of the risk of candidemia in the ICU [11].

In critically ill patients, the length of ICU stay before IC diagnosis is a major factor but it varies greatly. In one of the few meta-analyses examining the time between ICU admission and candidemia onset, Zhang et al. (2020) reported an average duration of 12.9 days, with some differences based on location [14]. The average hospitalization duration was 36.3 days, though the complexity of risk factors led to high variability in both the overall cohort and specific subgroup analyses.

Differentiating between prior *Candida* spp. colonization and the onset of invasive candidiasis can be difficult. For patients with endogenous colonization and risk factors, the typical time to develop IC is about 7–10 days [14]. *Candida* colonization is found in 5–15% of ICU patients at admission, increasing to 50–86% with longer ICU stays. However, only 5–30% of these colonized patients develop invasive candidiasis. Recent studies have shown even higher colonization rates at ICU admission, ranging from 27% to 70% [15,16,17].

Since multifocal colonization is an independent risk factor, ICU departments have implemented active surveillance by collecting samples from various body sites. These screening methods help identify patients at high and low risk [11,18]. In a multicenter study, Leon et al. (2006) observed that the mortality rate was higher among ICU patients with multifocal colonization (50.9%) compared to those with unifocal colonization (26.5%) [19].

Surgery is another significant risk factor, particularly when it involves the gastrointestinal tract, as the gut is a common site for *Candida* colonization. Additionally, surgical patients often have comorbidities that promote *Candida* colonization and the development of invasive candidiasis, including hypertension, diabetes, heart, respiratory, renal diseases, and malignancies. In a cross-sectional study of patients admitted to surgical ICUs, Tathler et al. reported that *Candida* spp. was detected more frequently in various samples after abdominal surgery than after other surgical procedures, and the overall incidence of invasive candidiasis was 1.06% [20].

Preexposure to broad-spectrum antibiotics is also recognized as a risk factor for invasive candidiasis, especially when combinations of more than two drugs are needed.

A nationwide Israeli study by Ben-Ami et al. (2012) found a significant link between bloodstream infections with fluconazole-resistant *Candida* spp. and prior exposure, either at the same time or sequentially, to antibiotics such as trimethoprim-sulfamethoxazole, carbapenems, clindamycin, and colistin [21]. In a multicenter matched case–control study, Poissy et al. identified some differences between the antibiotic exposure in ICU patients and non-ICU patients [11]. Aminoglycosides were an independent risk factor for candidemia only in the ICU patients and glycopeptides and nitroimidazoles were associated with candidemia only in non-ICU patients.

Septicemia caused by bacteria can create conditions that favor invasive fungal infections. In sepsis patients, resulting immunosuppression, compromised gut mucosa barrier, and treatments like antibiotics and invasive procedures all increase the risk of *Candida* spp. infections.

Acute kidney injury or other causes associated with renal failure in critically ill patients and the need to insert a venous catheter to initiate renal replacement therapy increase the risk of invasive candidiasis. Other related factors include contamination by *Candida* spp. and biofilm formation on various sites, such as dialysis machine circuits, hydraulic pipes, tread water, or other fluids like dialysate or carbonated solutions [22,23].

In acute liver disease, the need for invasive procedures, antibiotic use, and internal factors such as impaired phagocyte activity all raise the risk of *Candida* spp. infections.

For chronic liver diseases, the risk of developing infections with *Candida* spp. increases proportionally with severity scores. It is believed that most cases of invasive candidiasis in patients with end-stage liver disease occur while patients are already hospitalized in ICUs. Less than 12% of patients with advanced liver cirrhosis develop spontaneous peritonitis. In 3–10% of cases, this serious complication has a fungal cause, and the mortality rate is higher than in bacterial peritonitis.

For these patients with renal and hepatic diseases, the associated immune dysfunctions are contributors to invasive candidiasis occurrence [24].

The COVID-19 pandemic has led to increased admissions to intensive care units. During SARS-CoV-2 infection, a deficiency in the interferon pathway—crucial for combating *Candida* infections—has been observed. Additionally, studies show that the impaired immune response involves reduced release of key cytokines (IL-6, TNF, IL-1α, IL-1β) against *Candida* spp. Factors such as mechanical ventilation, diabetes mellitus, neutrophilia, and low hemoglobin are significant risks. The occurrence of candidemia in severe COVID-19 cases is notably higher—ranging from 0.8% to 14%—compared to patients without COVID-19 [25,26].

Oncology patients also have an impaired immune response, and their risk of invasive candidiasis increases when the integrity of the intestinal mucosa is disrupted, allowing local *Candida* spp. overgrowth and translocation into the bloodstream.

Several studies have linked candidemia to hematological malignancies such as acute leukemia, lymphoma, or myelodysplastic syndrome, as well as solid tumors like gastrointestinal and genitourinary cancers, with 30–50% of candidemia patients having underlying malignancies [27,28]. Oncology chemotherapy is also a key factor since some drugs can cause intestinal mucositis or significantly disrupt the balance between bacterial and fungal microbiomes, which can increase the pathogenicity of *Candida* spp.

Other vulnerable groups include patients receiving multiple blood transfusions or undergoing an immunosuppressive regimen for underlying autoimmune disease or vasculitis [29].

### 3.2. Genetic Factors

Research on Dectin-1, a β-glucan receptor, and its adapter CARD9 has significantly improved our understanding of candida infections. The Y238X polymorphism in the *Dectin-1* gene reduces its expression, impairing the recognition of β-glucans in *C. albicans* cell walls. This impairment decreases the release of key cytokines like IL-17 and TNF, which are essential for immune defense. Some studies indicate that this polymorphism may increase the risk of systemic invasion in critically ill patients who acquire *Candida* spp. infections during medical procedures. However, findings are inconsistent, highlighting the complex relationship between this genetic variation and susceptibility to different forms of candidiasis across various patient groups [30,31].

Loss-of-function mutations in CARD9 are linked to inherited susceptibility to invasive *Candida* infections, highlighting the role of genetic factors in disease risk.

In 2019, Glocker et al. was the first to document CARD9 deficiency in an Iranian consanguineous family, linking the Q295X mutation to chronic mucocutaneous candidiasis and increased susceptibility to various types of mycosis, including disseminated and cerebral forms [32].

### 3.3. Risk Factors in Newborns

Low-birth-weight newborns are also at risk of developing invasive *Candida* infections, as the colonization rate is relatively high in this group. Colonization can be acquired either vertically during vaginal delivery or horizontally in the neonatal intensive care unit environment, through healthcare providers’ hands, contaminated equipment, or intravenous preparations. Additional risk factors include a low Apgar score, mechanical ventilation, parenteral nutrition, delays in transitioning to enteral nutrition, prolonged antibiotic therapy, extended hospitalization, and severe coexisting conditions such as shock or disseminated intravascular coagulopathy.

The occurrence of invasive *Candida* infections decreases with higher birth weight and gestational age. During the first month in the neonatal intensive care unit, over 60% of very-low-birth-weight infants are colonized by *Candida* species, and invasive candidiasis occurs in 8 to 23% of these cases [33].

## 4. Clinical Manifestations in Invasive Candidiasis

Early and accurate diagnosis is crucial for effective treatment and patient recovery. However, diagnosing a *Candida* spp. infection is difficult because of the complex interactions with the host, its ability to mimic other conditions, and the lack of specific symptoms.

This pathology typically manifests in three forms: candidemia without deep-seated candidiasis, deep-seated candidiasis without candidemia (caused by direct inoculation or previous hematogenous spread), and candidemia followed by deep-seated candidiasis, which can affect various organs or submucosal tissues.

In ICU patients invasive candidiasis falls into three main categories: candidemia, intra-abdominal candidiasis (IAC) and possible invasive candidiasis, which is less common and has different clinical manifestations depending on the site of infection.

*Candida* sepsis

Candidemia has no specific symptomatology; the clinical manifestations can vary significantly. It might refer to fever alone, leukocytosis alone, the absence of organ-specific manifestations, or a wide spectrum of manifestations, including culture-negative septic shock.

Clinical manifestations of candidemia may frequently overlap with those of underlying conditions and are indistinguishable from those identified in bacterial infections, appearing as sepsis, septic shock, or multiorgan failure [34,35].

Intra-abdominal candidiasis

Among non-neutropenic, critically ill ICU patients, intra-abdominal candidiasis is the most common form of deep-seated candidiasis and occurs in the presence of certain risk factors such as: gastrointestinal perforation, anastomotic leakage, intra-abdominal drains and antibiotic treatment for more than 7 days [36,37]. The lack of clear criteria to differentiate between *Candida* colonization and true *Candida* infection makes early diagnosis difficult. This condition is reflected by various clinical syndromes. Vergidis et al. developed a schematic classification based on the anatomic sites of infection [38]. The main IAC manifestations are: primary peritonitis, secondary peritonitis, intra-abdominal abscesses, infected pancreatic necrosis and cholecystitis or cholangitis with *Candida* spp. Secondary peritonitis and abscesses are the most frequent and can originate from both the gastrointestinal tract and hepatobiliary/pancreatic sources.

Appropriate source control (drainage of infected material, surgical intervention to correct the perforation or anastomotic leakage) and immediate antifungal treatment with correctly adjusted doses are crucial for improved outcomes [39].

Possible invasive candidiasis

*Candida* endocarditis

Fungal endocarditis is a rare condition, representing 1–6% of all cases. It typically occurs during episodes of candidemia in patients with risk factors such as prosthetic heart valves, intravenous drug use, and central venous catheters. An echocardiogram is recommended when these risk factors are present along with symptoms suggestive of the condition.

Pneumonia with *Candida* spp.

Pneumonia caused by *Candida* spp. is extremely rare and can occur in immunocompromised individuals, such as those with disseminated conditions, those with cancer, or low-birth-weight infants, often due to hematogenous spread or microaspiration. Diagnosing this is challenging because it is difficult to confirm a link between *Candida* spp. presence and pulmonary symptoms, even if they are isolated from respiratory samples.

Esophageal candidiasis and upper respiratory colonization can mislead the diagnosis. Symptoms of pneumonia caused by *Candida* spp. are nonspecific; neutropenic patients might only present with fever. Radiological signs such as bronchopneumonia, opacities, abscesses, and cavitary lesions are also nonspecific, with nodules being the most common finding on CT scans [40,41].

*Candida* spp. can infect bones and joints through bloodstream seeding or other routes such as trauma, surgery, or injection drug use. Symptoms are often subtle, leading to delays in diagnosis, especially in cases of vertebral osteomyelitis. Importantly, even a single *Candida* colony found in a biopsy or aspirate is considered a sign of infection.

Central nervous system (CNS) candidiasis

This rare condition mainly affects the meninges, with higher incidence in premature neonates and AIDS patients with low CD4+ T lymphocyte counts.

These infections can occur through hematogenous spread or direct inoculation, often influenced by factors such as neurosurgery and recent use of antibiotics and corticosteroids. Besides meningitis and vascular issues, other clinical signs include cerebral micro abscesses, which are common complications in immunosuppressed patients with candidemia involving the CNS. Generally, symptoms include fever, meningismus, increased cerebrospinal fluid pressure, and specific neurological signs. Performing a lumbar puncture and cranial imaging is necessary when infection is suspected.

Ocular candidiasis

This can involve chorioretinitis and/or endophthalmitis in rare cases of candidemia. Symptoms may be minimal, so fundoscopic examinations are essential to assess the extent of eye involvement. In endophthalmitis, the risk of losing sight is high because it starts as chorioretinitis but can extend into the vitreous, leading to vitreous abscesses.

Candiduria

The presence of *Candida* spp. in urine is common, especially among hospitalized patients, with up to 90% of those with *Candida* UTIs being catheterized. Often, it indicates colonization or contamination, particularly if asymptomatic. In patients with urinary obstructions or undergoing procedures, it can promote candidemia.

Once microbiological confirmation is obtained, physicians should identify and evaluate key risk factors such as renal dysfunction, immune deficiencies, and the extent of involvement in the genitourinary system to guide effective treatment decisions [42].

Manifestations in neonates

Neonates can develop two primary types of invasive candidiasis. The first, congenital candidiasis, occurs when the infection is transmitted from the mother before or during birth and is often linked to multiple skin lesions. The second type is postnatal invasive candidiasis, which results from contamination of central venous catheters and causes candidemia in 70–95% of such cases.

The symptoms of neonatal invasive candidiasis often resemble those of bacterial late-onset infections. It presents with nonspecific signs such as apnea, respiratory distress, bradycardia, temperature instability, feeding difficulties, decreased spontaneous activity, or lethargy. *Candida* species can spread through the bloodstream or create septic emboli, leading to infections in deep tissues or forming fungal masses in multiple organs.

The central nervous system is frequently affected, resulting in conditions such as meningitis, encephalitis, or sometimes ventriculitis and brain abscesses. Neurological issues like cerebral palsy, visual and hearing impairments, and varying degrees of neurodevelopmental delays are more often seen in preterm infants with IC than in those without.

Renal involvement can present as cystitis or more severe conditions such as parenchymal infiltration and obstructive uropathy caused by fungal masses.

Additionally, endocarditis, though rare, is a serious complication linked to prolonged candidemia and central venous catheter use. Other uncommon manifestations include ocular infections and osteomyelitis or abscesses in the liver and spleen [43].

## 5. Definitions of Invasive Candida Infections Currently in Use

### 5.1. Invasive Candidiasis

The European Organization for Research and Treatment of Cancer (EORTC) and the Mycoses Study Group Education and Research Consortium (MSGERC) define two categories of invasive fungal disease (IFD): “proven” and “probable.”

The term “proven IFD” applies to all hosts regardless of immunocompromised status and is defined by the detection of a fungus in blood culture, histological examination, or culture of tissue from sterile sites.

In contrast, the “probable IFD” is context-dependent, requiring an interaction of three elements: the host factors that indicate a patient is at risk, the clinical signs that match the disease, and the mycological evidence gathered not only from cultures and microscopy but also from indirect tests such as antigen detection and polymerase chain reactions.

This structured approach is designed to help in formulating the diagnosis of IFD in clinical settings, especially in ICU environments, but challenges still arise during its implementation [44,45].

In the ICU, candidemia is the most common confirmed invasive fungal infection (IFD). Blood samples are collected in 10 mL blood culture bottles for both aerobic and anaerobic testing. To reliably confirm candidemia, clinicians gather two pairs of blood culture bottles—preferably up to four pairs within 24 h—thus achieving detection rates above 90%.

### 5.2. Catheter-Associated Candidemia

Approximately 40–50% of candidemia cases are linked to intravenous catheters, which are commonly used in ICU patients [46]. Over recent decades the use of intravascular devices has extended in non-ICU settings too. Underlying medical conditions like malignancies, solid organ transplantation, hematopoietic stem cell transplantation or myeloablative therapies often require insertion of CVCs.

The European Centre for Disease Prevention and Control (ECDC) definition of catheter-related bloodstream infection states that it occurs within 48 h before or after catheter removal. This diagnosis is confirmed by a positive blood culture identifying the same microorganism found in a quantitative culture from a catheter tip (≥10^3^ UFC/mL), a semiquantitative catheter culture (>15 colony-forming units), or a positive culture from pus at the insertion site [46].

Another difficult situation occurs when candidemia is suspected in patients with central venous lines. In this case, blood samples are taken from both the central line and a peripheral site to distinguish between catheter-associated and non-catheter-associated infections. Comparing the time to positivity or the number of colony-forming units from blood collected via the catheter versus peripheral circulation helps make this distinction.

The early removal of the CVC and the use of echinocandins in the first-line therapy are the main recommendations for best clinical practice provided by the guidelines of the Infectious Diseases Society of America (IDSA) and the European Confederation of Medical Mycology (ECMM). Multicenter studies demonstrated that the lack of CVC removal within 48 h after confirmed candidemia and no initiation of effective antifungal therapy are independent factors associated with 30-day mortality [47,48]. The catheter removal is effective either directly or indirectly. In situations when the catheter is not the primary source of infection, a persistent candidemia increases the risk of catheter colonization and subsequently perpetuating the infection [49].

### 5.3. Deep-Seated Candidiasis

Intra-abdominal candidiasis remains a major problem as the most common form. The diagnostic approach starts with the identification of patients with certain risk factors followed by collection of intra- or perioperative samples (fluid or tissue), samples from freshly placed drains (<24 h) or other clinically relevant samples [49,50,51]. However, the clinical significance of *Candida* presence is controversial as mixed infection with bacteria, particularly *Enterococcus* and *Enterobacteriaceae*, is frequent. Identification of *Candida* spp. in the blood culture remains the gold standard for diagnosing invasive candidiasis, including intra-abdominal candidiasis, though its sensitivity is modest [49]. Serological determination of biomarkers is also considered, although their contribution to diagnosis is under discussion. Leon C et al. identified 1,3-ß-D-glucan (BDG) in combination with anti-germ tube antibodies (CAGTAs) as the most suitable biomarkers that can be used in diagnosis of IC in patients with acute abdominal complications [37].

IAC requires early source control and immediate antifungal treatment. In a retrospective study, Verghidis et al. defined early source control as an intervention performed within 5 days from the moment of collecting the samples in which *Candida* spp. were identified [38]. Abscesses were more likely to be treated by percutaneous drainage and secondary peritonitis by surgical interventions. For a long time, for the antifungal treatment there was no sufficient data to recommend a preferred agent and the time of initiation was more important for patient’s prognosis [38]. Currently, the main guidelines elaborated by ESCMID, ECMM and IDSA mention with high-quality evidence echinocandins as first-line treatment for invasive candidiasis without specific consideration for IAC [50]. As the acquired echinocandin resistance of non-albicans species, especially *Candida glabrata*, threatens to expand, Peman et al. concluded that echinocandins should be used in peritoneal candidiasis if the patient is hemodynamically unstable, azole therapy was previously used or the isolated *Candida* spp. from peritoneal fluid is fluconazole-resistant [49].

Other deep-seated candidiasis is difficult to diagnose because it requires direct sampling from deep infection sites, which is done in less than 50% of patients due to significant risks. It can also be contraindicated in patients with severe underlying conditions. Challenges include small sample sizes, low fungal cell concentrations, and uneven distribution—all of which can hinder accurate diagnosis.

## 6. Microbiological and Serological Diagnosis of Invasive Candidiasis

### 6.1. Culture-Related Methods

**Figure 2:**
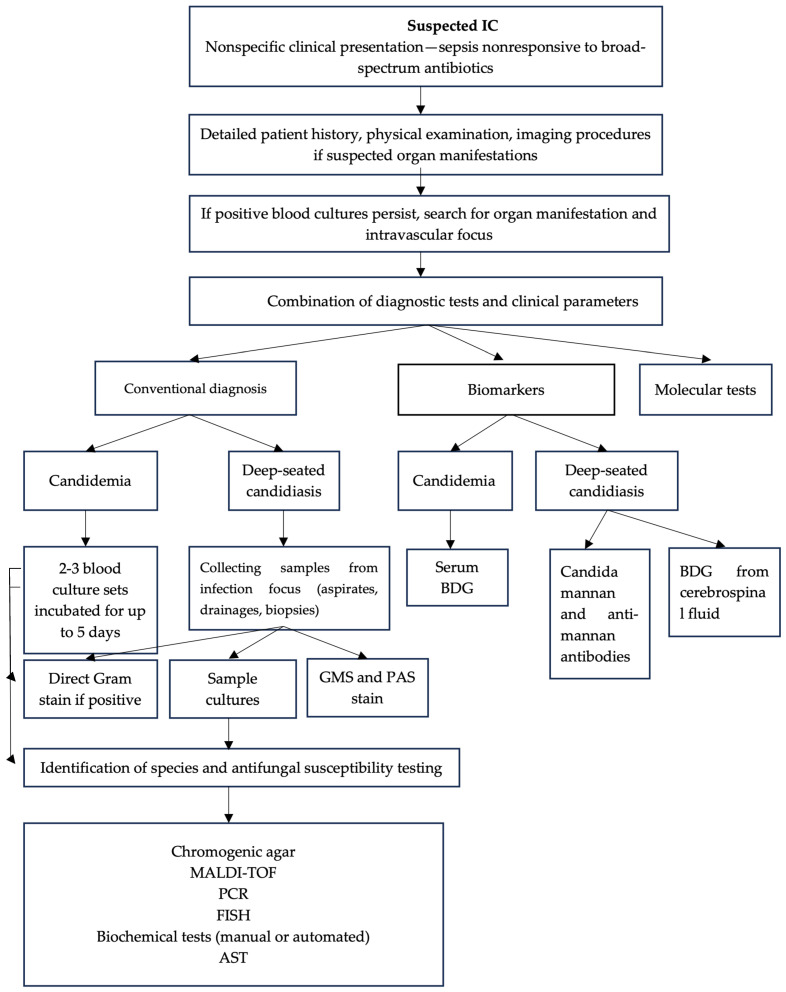
Invasive candidiasis diagnostic algorithm according to the guidelines of ECMM/ISHAM and ASM [50].

Blood culture is regarded as the gold standard for diagnosing invasive infections caused by *Candida* spp. (Figure 2). However, it has two major drawbacks. The first is a long turnaround time—ranging from 1 to 7 days or more—for a culture to be reported positive, plus an additional 2 or 3 days for species identification and antifungal susceptibility testing. The second issue is that low-concentration candidemia (≤1 CFU/mL) or deep-seated *Candida* infections without candidemia are common situations where blood cultures may fail to detect the fungus [51,52]. According to recent estimates provided by Denning et. al, blood cultures become positive in approximately 40% of invasive candidemia cases [4].

In the absence of a positive blood culture, diagnosis is challenging, and clinicians should consider using a combination of complementary methods like non-culture diagnostics, especially when patients exhibit signs of systemic infection (Table 1).

Conventional methods for identifying *Candida* species from positive cultures depend on morphological and biochemical features or characteristics on chromogenic media.

For example, CHROMagar is recognized for its ability to distinguish among common *Candida* species. Specifically, for *C. auris*, a salt/dulcitol enrichment broth containing 10% NaCl promotes its growth while inhibiting other species. Accurate identification occurs in about 76% of cases. Although these methods cannot identify less common *Candida* spp., they remain valuable in diagnostics because they can distinguish mixed cultures.

Automated biochemical identification systems enhance etiological diagnosis and often offer antifungal susceptibility testing as well. VITEK 2 identifies *Candida* spp. with sensitivity and specificity exceeding 95%.

The Becton Dickinson Phoenix Yeast ID Panel demonstrated 94.4–97.3% accuracy in identifying *Candida* isolates and required 4–15 h for identification. The Remel RapID Yeast Plus System also has high accuracy (95.3%), but the manual method requires a minimum of 52 h [53].

However, these traditional methods are becoming outdated due to modern techniques such as Matrix-Assisted Laser Desorption–Ionization Time-Of-Flight (MALDI-TOF) mass spectrometry. This technology allows for rapid identification of different species, including *C. auris*, with results available in 10 to 15 min after cultures are isolated on artificial media. The method has a sensitivity ranging from 56 to 73%. MALDI-TOF MS also can provide insights into resistance patterns to antifungals like fluconazole, caspofungin, and anidulafungin in species such as *C. albicans* and *C. glabrata*.

Polymerase chain reaction (PCR) technology is also regarded as a groundbreaking tool in diagnostic microbiology, especially for identifying pathogens like *Candida* spp. PCR greatly speeds up diagnosis and can detect as few as 10 CFU/mL. However, the test may fail to identify *Candida* spp. in cases with low fungal load, such as candidemia originating from an abdominal site, or in patients with neutropenia, recent major surgery, end-stage liver disease, or on renal replacement therapy.

When performed on positive blood cultures, PCR achieves sensitivities of 90–95% and specificities of 90–92%, but this high effectiveness depends on strict aseptic blood collection to prevent contamination and false positives.

The BioFire FilmArray BCID assay is a multiplex PCR that detects 24 organisms, including 19 bacteria and five of the most common *Candida* species, i.e., *C. albicans*, *C. glabrata*, *C. parapsilosis*, *C. tropicalis*, and *C. krusei*, from positive blood cultures. The sensitivity is 100%, and results are available in one hour. The updated panel version—BCID2—also identifies *C. auris* [54].

The T2*Candida* Panel is a nanodiagnostic test based on PCR technology currently discontinued by the manufacturer. Sensitivity and specificity for identifying *Candida* species in whole blood are 89–91% and 98–99%, respectively. Results are available within 3–4 h and can identify *C. albicans*/*C. tropicalis*, *C. parapsilosis*, and *C. krusei*/*C. glabrata*, although the test cannot precisely differentiate among these species. A notable advantage is its low detection threshold of 1 CFU per 60 mL of blood (routinely obtained from three 20 mL samples) [54].

### 6.2. Non-Culture-Based Methods

Serum biomarkers: The low sensitivity of traditional “gold-standard” methods remains an ongoing challenge, requiring more approaches in diagnostic strategy.

Alternative laboratory methods that do not involve culture or histopathology are available and should be used to improve diagnostic accuracy and assist in detecting invasive fungal diseases before microbiological confirmation.

The detection of 1,3-β-D-glucan, a component of the cell wall in *Candida* spp., functions as a biomarker for invasive fungal infections, with the test exhibiting a sensitivity of 92% and a specificity of 81%.

Its reliability is influenced by the presence of 1,3-D-glucan in fungi like *Aspergillus* and *Pneumocystis jirovecii*, which can lead to diagnostic confusion. However, the test’s high negative predictive value effectively rules out invasive candidiasis. The FDA has approved this test for diagnosing invasive fungal infections and includes it as part of the mycologic evidence for identifying probable invasive candidiasis cases [52]. Moreover, several studies examined the potential of 1,3-β-D-glucan assays in predicting the clinical outcome of candidemia. A 1,3-β-D-glucan concentration below 416 pg/mL during antifungal therapy was identified as a predictor of therapeutic success with the condition of no iatrogenic contamination with certain drug formulas that might contain 1,3-β-D-glucan, thus temporarily interfering with the result [55].

Mannan is another crucial cell-wall antigen specific to *Candida* species, and it, along with anti-mannan antibodies, has been suggested as a biomarker. These tests demonstrate a sensitivity of only 55% and a specificity of 65% in detecting invasive candidiasis, even when combined [53]. However, several studies report that the sensitivity of the mannan assay varies among *Candida* species, being highest for *C. albicans*, followed by *C. glabrata* and *C. tropicalis*. The assay shows lower sensitivity for *C. parapsilosis* and *Pichia kudriavzevii*, likely because these species produce smaller amounts of mannan. An additional important issue is that elevated anti-mannan antibody levels in the absence of mannan detection can be misleading, as they are also found in patients with prior *Candida* infections, especially those who are immunocompromised or have autoimmune diseases. Currently, neither testing method has received FDA approval for diagnosing invasive candidiasis [56].

However, several studies have shown that integrating BDG assays with mannan detection assays improves diagnostic accuracy. While BDG is nonspecific, positive results for mannan or anti-mannan antibodies suggest a *Candida* infection, whereas negative results may indicate infection from other fungal sources. The European Society of Clinical Microbiology and Infectious Diseases has elaborated recommendations for serial testing of mannan antigen and anti-mannan antibody for the diagnosis of deep-seated candidiasis [50].

When an invasive candidiasis is suspected and diagnostic tests like those above are performed, a clinician should be aware of the possible pitfalls that can lead to a premature conclusion. Key strategies that should be considered are: screening for possible fungal metastatic foci, addressing the source (example: removing a contaminated catheter), no treatment delay until the culture becomes positive, and not depending on a single negative biomarker value to exclude the diagnosis.

## 7. Predictive Scores—Risk Assessment

The limitations of diagnostic tests has led to the creation of scoring systems for IC risk assessment (Table 2). The role of these clinical predictive models is to identify and categorize patients and assist in clinical decision-making and definitely not replace the diagnostic methods for invasive candidiasis. The first and most frequently used clinical prediction model is *Candida* score and it will be further discussed.

It is a research-based score designed to identify patients at high risk of developing invasive candidiasis. It was proposed in 2006 by Leon et al. The database included 1107 adult ICU patients hospitalized in 36 ICUs across Spain, France, and Argentina and was part of the Estudio de Prevalencia de CANdidiasis project [19].

As the concept of severe sepsis was later removed, the current *Candida* score was updated to the Sepsis 3.0 definition. The assessment of organ dysfunction using the SOFA score has proven to be clinically helpful; as a result, the new *Candida* score’s accuracy is improved. *Candida* score was tested in numerous studies. An example is a prospective cohort study which concluded that “*Candida* score” was found to be reliable in differentiating ICU patients who might benefit from early antifungal treatment from those with unlikely invasive candidiasis, as the incidence of invasive candidiasis was indeed proportional to the *Candida* score [57].

Later, a cutoff value (>3) for prescribing prophylactic antifungals was decided when applying *Candida* score, but using this assessment in everyday practice may lead to unnecessary or excessive use of antifungals [57,58].

Vazquez et al. described a single-center experience of starting empiric or early presumptive antifungal therapy when the following criteria were met: patients at high risk of IC, a *Candida* score of ≥3, more than 72 h on broad-spectrum antibiotics, and an initial positive BDG assay. If the BDG assay remained positive after 48 h, antifungal therapy was continued. If the test was negative, antifungals were stopped. This approach helped reduce unnecessary therapy, cutting usage by 50% [39].

Given the limited evidence supporting empirical antifungal therapy in patients with severe sepsis or septic shock, the low rate of blood culture positivity, and the concern that any delay can worsen prognosis, decisions to start treatment are often made. However, current predictive rules have some disadvantages. In a systematic review, Giacobbe et al. evaluated the contribution to IC diagnostic of these tools across 16 studies [58]. Despite the heterogeneity of patient categories and different prevalences of IC, all the predictive models showed a high negative predictive value (NPV) and a low positive predictive value (PPV) for the diagnosis of IC. Subsequently, the expert recommendations are to use these models rather to rule out the category of patients at low risk of IC. This is still a valuable finding as it exempts these patients from empirical antifungal therapy.

Other disadvantages are: complicated use in some cases, and limited prospective validation. For example, empirically treating all patients with sepsis with antifungals and automatically assigning a *Candida* score of at least 2 in the presence of one additional risk factor (surgery, total parenteral nutrition, or multifocal *Candida* colonization) might seem excessive to some intensivists and need further validation studies.

A predictive model is considered useful for clinicians if it can be readily applied at other institutions, thus having generalizability and good accessibility.

In 2018, to standardize clinical practice, the European Confederation of Medical Mycology (ECMM) created the European Confederation of Medical Mycology Quality of Clinical Candidemia Management (EQUAL) *Candida* score. This is the first tool that aligns with current guidelines by assessing the quality of indicators used for diagnosis, treatment initiation, and follow-up. It evaluates the success rate of initial empirical treatment with echinocandins and de-escalation to fluconazole if susceptibility is confirmed [59]. Many studies have already assessed this score’s impact on mortality. While some (e.g., Zakrem et al., Salmanton-Garcia et al.) found a significant difference in EQUAL score values between patients with CVC who survived the ICU and those who did not, other studies (Pinto-Magalhães et al.) reported no significant association [60,61].

**Table 2:** Predictive models for invasive candidiasis for ICU and non-ICU patients.

Models	Variables	Cutoff Point	Sensitivity/ Specificity	Strength	Weakness
**ICU models**					
*Candida* score (2006) [19]	1 point = total parenteral nutrition 1 point = preexisting surgery 1 point = multifocal *Candida* colonization 2 points = severe sepsis	≥3	81%/74%	Can be applied in prevention and treatment decisions as IC (if present) was defined after at least 7 days of ICU stay	Patients with neutropenia were not included so this model cannot be applied to this category *Candida* colonization included as a predictor might limit the model application in settings without surveillance culture protocols
Ostrosky-Zeichner Rule (2007) [59]	Systemic antibiotic use (days 1–3) OR CVC presence (days 1–3) AND at least 2 of the following: Total parenteral nutrition (days 1–3) Dialysis (days 1–3) Major surgery (days -7 to 0) Pancreatitis (days -7 to 0) Steroid therapy (days -7 to 3) Other immunosuppressive agents (days -7 to 0)	Not specified	34%/90%	Can be applied in prevention and treatment decisions as IC (if present) was defined after 4 days of ICU stay Can identify patients at high risk of fluconazole high-risk failure in candidemia	Cannot be applied to patients with systemic antifungal treatment prior to day 4 of ICU stay
Nebraska Medical Center Rules (2011) [62]	Any broad-spectrum antibiotic use Central venous catheter (D1 to D3), abdominal surgery (D-7 to D3) Immunosuppressants (D-7 to D0) Total parenteral nutrition (D1 to D3) Mean pre-ICU length of stay	<2.45	84%/60%	Can be applied in prevention and treatment decisions as IC (if present) was defined after 4 days of ICU stay	Derived from a single-center retrospective chart review study and validated in the same population Cannot be applied to patients with systemic antifungal treatment prior to day 4 of ICU stay
Corrected Pittet colonization index [63]	Calculated as the ratio of the number of sites with high colonization (>10^5^ CFU) to the number of cultured sites	≥0.4	100%/100%	High positive predictive value in discriminating the colonized from infected patients if 3 variables were documented: length of previous antibiotherapy, APACHE II score of illness severity and intensity of *Candida* spp. colonization	The cost and processing time might be increased as it requires quantitative cultures from a large number of sites
Shanin et al. model (2016) [64]	Pancreatitis CVC number Highest heart rate in the first 24 h: >100 beat per minute Fungal colonization	Not specified	40.5%/84.5%	Huge sample size of admissions to a large number of critical care units across the UK (96 critical care units) Rigorous statistical methods (logistic regression with robust standard error) to build model, including adjusting for clustering within ICU	Low number of events per variable because the observed rate of IC was approximately half of that anticipated by authors Performance might be lower when translating the model to different geographical settings Cannot be applied to patients with IC or receipt of systemic antifungals 7 days prior to admission Cannot be applied to patients with hematological malignancies or recent solid organ transplant
The AMI risk assessment tool (2025) [65]	Mechanical ventilation Types of antibiotics used Immunosuppressive drugs			Ability to predict if ICU sepsis patients will develop IC	Cannot be applied to patients with non-infectious diseases or to patients without evaluation of SOFA score or SOFA score <2
**Non-ICU models**					
Shorr et al. model (2009) [66]	Age under 65 years Temperature of 98 °F or less Cachexia Previous hospitalization in the last 30 days Transferred from another healthcare facility Mechanical ventilation at admission	≥1	90%/28.9%	Variables routinely available at presentation No need for cultures or other tests to confirm the presence of colonization, sepsis, or other conditions	Limited population (included only patients with candidemia diagnosed within two days of admission) Cannot be applied for suspected nosocomial candidemia Lack of data about some specific risk factors in candidemia (prior exposure to antibiotics, recent surgery, total parenteral nutrition on admission, CVC placed)
Falcone et al. model (2017) [67]	Severe sepsis/septic shock Recent Clostridium difficile infection Diabetes mellitus CVC Total parenteral nutrition Chronic obstructive pulmonary disease Previous antibiotherapy Concomitant glycopeptide therapy Immunosuppressive therapy Peripherally inserted central catheter	>3	87%/83%	The first to take into consideration Clostridium difficile infection as these two infections share some similar risk factors	Limited population (included only non-ICU patients from medicine wards and non-neutropenic patients) Cannot be applied to patients with any malignancies
Sozio et al. model (2018) [68]	Total parenteral nutrition CVC Peripherally inserted central catheter Previous hospitalization antibiotherapy Antibiotherapy during hospitalization Neurological disability—previous hospitalization in the last 3 months	≥4	84%/76%	Highlights the use of antibiotics as risk factor	Limited population (included only non-ICU patients from medicine wards with positive blood cultures)
Ruiz Rougomez et al. model (2018) [69,70]	Male sex Steroid therapy Total parenteral nutrition Urinary catheterization Previous antibiotherapy	≥7	79.2%/82.6%	The first to include steroid therapy as a predictor of IC	Limited population (included only non-ICU patients who were non-critically ill, non-neutropenic and without surgical interventions)

Overall, more strategies are needed to improve the implementation of these models. Digital tools, such as a scoring calculator, should be developed, minimizing resource consumption through adaptation to available technical resources. The workflow should not require additional time from personnel, and regular benchmarks should be conducted externally.

## 8. Concepts and Candidate Biomarkers Derived from *Candida* spp. Mechanisms of Invasiveness

In cases of immunosuppressive diseases or any disruption of the normal microbiota, some *Candida* spp. develop invasive capabilities (e.g growing hyphae), becoming human pathogens by disrupting their colonization sites and potentially affecting any organ.

The primary functions of hyphae include actively penetrating host epithelial cells and inducing endocytosis.

The role of hyphae in virulence is intricate, involving the production of specific adhesion molecules or toxins. The adherence to host cells is facilitated by numerous specialized proteins called adhesins, with Als family (Als1–7 and Als9) being dominant. Als3, exclusive to *C. albicans* strains, is especially important because of its critical role in adhesion and biofilm formation. It allows adherence to a wide range of substrates, including E-cadherin on epithelial cells, N-cadherin on endothelial cells, fibronectin and many others. [71].

Another hyphae-specific adhesin supporting Als3 in mature biofilm formation is the hyphal wall protein Hwp1, which interacts with similar host cell substrates [72].

Recent studies have examined the potential of these proteins as diagnostic targets (Table 3). For example, Diez et al. (2021) developed enzyme-linked immunosorbent assays (ELISAs) to detect antibodies against Als3, Hwp1, and Met6, aiming to find potential biomarkers for invasive candidiasis in both immunocompetent and immunocompromised patients [73].

In immunocompetent patients, the sensitivity for detecting anti-Als3 antibodies was 83%, while it was lower (75%) in immunocompromised patients. Conversely, the detection of anti-Met6 antibodies demonstrated a high sensitivity of 92% for assessing the risk of IC in immunocompromised individuals [74]. Met6 is a cell-surface protein that functions as a methionine synthase. Met6 becomes activated under thermal stress or to reduce homocysteine toxicity by converting homocysteine into methionine.

The high negative predictive value of anti-Met6 antibodies (97%) was seen as a potential guide for early antifungal treatment or, alternatively, for de-escalation if these antibodies are absent. In a 2023 study, Bromuro et al. included anti-Als3 or anti-MP65 antibody levels in a multivariable logistic model along with other classical variables and reported that these antibodies were significantly associated with lower mortality at 30 days [74].

The immune response to hyphal wall protein 1 (Hwp1), a mycelial-phase antigen of *C. albicans*, is also seen as a potential serodiagnostic marker for invasive candidiasis. Initially identified in *C. albicans*, this antigen is also produced by other *Candida* species such as *C. tropicalis*, *C. glabrata*, *C. parapsilosis*, and *Pichia kudriavzevii*. The test shows variable sensitivity and specificity, performing better in cases of candidemia but with a risk of false positives in bacterial coinfections. Further research is needed to understand the kinetics of these antibodies, as serological responses typically become positive after more than 15 days. Its usefulness could be improved, especially for ruling out candidemia in treated patients and avoiding unnecessary antifungal treatments.

The surface adhesins also activate the subsequent members of the *Candida* proteome. It was reported that a specific moonlighting protein called enolase is activated by Als3.

Enolase plays a crucial role in fungal metabolism, but mainly helps facilitate pathogen migration through host tissues. Infection progression is supported by excess kinins produced from enolase interactions with plasma factors: kininogen, factor XII, and prekallikrein. It also promotes adhesion to extracellular matrix proteins which are associated with rapid humoral responses in systemic candidiasis patients.

The extracellular form of Eno1 is expressed by cells that form biofilms. It helps in sticking to substrates like silicone and polyvinyl chloride, which are common biocompatible materials used in medical devices such as valves, venous catheters, and orthopedic prostheses [75]. During invasive candidiasis, enolase appears in the blood of patients and can be used as a biomarker for diagnosis. Some studies have designed ELISA tests to detect anti-Eno1 antibodies [76].

Candidalysin is the first identified peptide with highly potent cytolytic activity released by the hyphal structures of *C. albicans*. According to Kasper et al., it is directly involved in the early destabilization of the macrophage membrane. Candidalysin likely plays a role in systemic infection, facilitating the translocation of fungi across the gastrointestinal epithelium. It has also been found that other species, such as *C. dubliniensis* and *C. tropicalis*, produce candidalysins that surprisingly exhibit more potent cytolytic activity [77]. In a 2025 study, Lou T et al., provide an indirect ELISA method for detecting anti-candidalysin IgG levels. Their findings suggest that this assay can effectively discriminate colonization from invasive infection at non-sterile sites as the antibody levels are responsive to deep-seated candidiasis. High levels were discovered in patients with positive stool, urine, sputum and blood cultures [78].

**Table 3:** Candidate biomarkers for invasive candidiasis (*none currently validated for diagnostic use).

Fungal Cell Component	Functions	Candidate Biomarkers/Diagnostic Procedures	State of Validation	Studies with Clinical Isolates from Patients with Fungal Infections
Als3 (morphology- dependent adhesin)	- Adherence to broad range of substrates (E-cadherin, N-cadherin, fibronectin, type IV collagen, laminin, EGFR, HER2, kininogen, fibrinogen, plasminogen) - A potent invasin	- Specific antibodies (ELISA)	Prototype procedures; not commercially available	Diez et al. (2021) [73] Size of cohort: 293 patients Se = 83% in immunocompetent patients, 75% in immunocompromised patients Sp = 63% in immunocompetent patients, 74% in immunocompromised patients AUC = 0.789/0.823
Hwp1 (hyphal wall protein 1)	- Hyphae-specific adhesin - Biofilm formation	- Specific antibodies (ELISA) - Hwp1 expression (qualitative RT-PCR)	Prototype procedures; not commercially available	Diez et al. (2021) [73] Size of cohort: 293 patients Se = 66% in immunocompetent patients, 75% in immunocompromised patients Sp = 75% in immunocompetent patients, 49% in immunocompromised patients AUC = 0.715/0.650
Met 6 (methionine synthetase)	- Metabolic pathways	- Specific antibodies (ELISA)	Prototype procedures; not commercially available	Diez et al. (2021) [73] Size of cohort: 293 patients Se = 77% in immunocompetent patients, 92% in immunocompromised patients Sp = 49% in immunocompetent patients, 74% in immunocompromised patients AUC = 0.674/0.892
Eno 1 (Enolase 1)	- Adherence to host matrix proteins like fibronectin, laminin and vitronectin - Important role in glycolysis pathways	- Specific antibodies (direct ELISA, sandwich ELISA, lateral flow immunoassay, serological proteome analysis—SERPA)	Available for research use only	He Zheng-Hin et al. (2016) [76] Size of cohort: 88 patients Se = 86.8% Sp = 90% AUC = 0.907
Candidalysin	- Hyphal-specific extracellular peptide - Facilitates translocation across gastrointestinal epithelium	- Specific antibodies (indirect ELISA)	Prototype procedures; not commercially available	Luo T et al. (2025) [78] Size of cohort: 121 patients Se = 80.0% Sp = 73.3% AUC = 0.818

*Candida* spp. exhibit numerous pathological traits, with more still to be identified. Novel biomarkers are continually being investigated in efforts to obtain better sensitivity and specificity, as well as good reproducibility, not only in reference laboratories or tertiary centers but also in all healthcare facilities.

## 9. Conclusions

Early suspicion and diagnosis of IC are crucial due to the high morbidity and mortality rates in high-risk patients. No definitive diagnosis is possible without confirmatory cultures, which are considered the “gold standard.” The time from sample collection to achieving a positive result can be as long as five days. Additional identification methods take varying amounts of time, and these delays can impact survival rates in IC. This is the primary challenge in IC diagnosis. Recently, molecular amplification techniques targeting various genetic sequences have allowed for the direct detection of many fungal species from collected specimens. Additionally, new biomarkers—such as fungal components or human antibodies—are being introduced or studied to identify fungal invasion before cultures become positive. Searching for more biomarkers is vital in clinical practice, especially for pre-symptomatic detection and real-time response markers. Understanding their kinetics and clearance should inform fungal treatment, help determine its duration, and predict potential relapse. The future of ICU diagnostics relies on multimodal strategies, including pathogen detection through multiplex panels, host response analysis, and clinical algorithms. Moreover, alongside risk stratification, frequent monitoring of circulating *Candida* spp. within each healthcare facility is necessary.

## Figures and Tables

**Table 1:** Overview of current diagnostic techniques for IC. References: [52,53,54].

Tests	Turnaround Time	Sensitivity	Specificity	Notes
**1. Performed on positive blood culture**				
Culture	2–4 days	21–71%	N/A	FDA-approved Allows susceptibility testing
Real-time, multiplex PCR (e.g., FilmArray^®^ BCID2 Panel, CandID^®^, Fungiplex^®^ *Candida*, LightCycler^®^ SeptiFast, Magicplex Sepsis^®^)	1–6 h	90–95%	90–92%	Not FDA-approved Culture-dependent *Candida* PCR should detect the most prevalent species and especially those associated with antifungal resistance (e.g., *N. glabratus*, *P. kudriavzevii* and *Candidozyma auris*)
T2*Candida* Magnetic Resonance	3–5 h	91%	99%	FDA-approved Detects the five major species: *C. albicans*, *C. krusei*, *C. tropicalis*, *C. parapsilosis*, and *C. glabrata* in whole-blood specimens collected in K2EDTA tubes; discontinued by manufacturer
**2. Performed on whole blood**				
1,3-β-D-glucan (e.g., Fungitell^®^, Fungitec-G^®^, Dynamiker Fungus assay^®^)	1 h	92%	81%	FDA-approved Blood collected directly from the vein in serum tubes Positive results can occur in other fungal infections
β-D-glucan+ procalcitonin	1 h	96%	98%	Blood collected directly from the vein in serum tubes Positive results can occur in other fungal infections
Mannan and anti-mannan IgG tests (e.g., Platelia *Candida* Ag-Plus and Ab-Plus^®^)		55%	65%	Not FDA-approved; does not discriminate between colonization and invasion
*C. albicans* germ tube antibody (CAGTA) assays (e.g., Vircell kit^®^ and VirClia IgG Monotest^®^)		42–96%	54–100%	CAGTA assay does not identify the fungal genus, thus confining the possibility of prescribing targeted antifungals in practice

## Data Availability

No new data were created or analyzed in this study. Data sharing is not applicable to this article.

## Abbreviations

The following abbreviations are used in this manuscript:

BDG	1,3-β-D-glucan
IC	Invasive candidiasis
ICU	Intensive care unit
FDA	Food and Drug Administration
